# Author Correction: Estimating the optimal age for infant measles vaccination

**DOI:** 10.1038/s41467-025-59082-w

**Published:** 2025-04-23

**Authors:** Elizabeth Goult, Laura Andrea Barrero Guevara, Michael Briga, Matthieu Domenech de Cellès

**Affiliations:** 1 Infectious Disease Epidemiology group, Max Planck Institute for Infection Biology, Charitéplatz 1, Campus Charité Mitte, Berlin, Germany; 2 Charité—Universitätsmedizin Berlin, Berlin, Germany; 3 Institute of Public Health, Charité—Universitätsmedizin Berlin, Berlin, Germany; 4 Department of Biology, University of Turku, Turku, Finland; 5 PandemiX Center of Excellence, Roskilde University, Roskilde, Denmark; 6 Charité Centre for Global Health, Charité—Universitätsmedizin Berlin, Berlin, Germany

**Keywords:** Viral infection, Computational models, Epidemiology, Health policy

Correction to: *Nature Communications* 10.1038/s41467-024-53415-x, published online 15 November 2024

In this article the affiliation ‘Charité—Universitätsmedizin Berlin, Berlin, Germany’ for Elizabeth Goult was missing. The original article has been corrected.

