# Supplementary material for: Estimating the optimal age for infant measles vaccination
Source: Nat Commun. 2024 Nov 15;15:9919. doi: 10.1038/s41467-024-53415-x (PMC11568136; doi:10.1038/s41467-024-53415-x)
Supplement: Supplementary file 1 — Supplementary Information [file 41467_2024_53415_MOESM1_ESM.pdf]

Estimating the optimal age for infant measles vaccination

Supplementary material

Elizabeth Goult[1,*] Laura Andrea Barrerro Guevara[1,2]
Michael Briga[1,3,4,5] Matthieu Domenech de Cellès[1]

1. Infectious Disease Epidemiology group, Max Planck Institute for Infection Biology, Charitéplatz 1, Campus Charité Mitte, Berlin, Germany
2. Institute of Public Health, Charité—Universitätsmedizin Berlin, Berlin, Germany
3. Department of Biology, University of Turku, Turku, Finland
4. PandemiX Center of Excellence, Roskilde University, Roskilde, Denmark
5. Charité Centre for Global Health, Charité—Universitätsmedizin Berlin, Berlin, Germany

∗ Corresponding author, email: goult@mpiib-berlin.mpg.de

Contents

1 Description of the model of measles transmission and vaccination 3
1.1 Protected by maternal antibodies 3
1.2 Susceptible 3
1.2.1 Incorporating vaccination 4
1.2.2 Susceptible equations 5
1.3 Infected 5
1.4 Recovered 6
1.5 Vaccinated susceptible 6

2 Supplementary tables 7

3 Supplementary figures 8

List of Tables

1 Delay quantiles from high income countries. 7
2 Model parameters. 8
3 Historical mean ages of infection. 9
4 Impact of transmission level on optimal age. 9
5 Impact of MCV1 coverage on optimal age. 10
6 Impact of MCV1 coverage on LMIC optimal age. 10
7 Impact of demographic type on LMIC optimal age. 11

List of Figures

1 MCV1 VE estimate countries. 11
2 MCV2 VE. 12
3 MCV1 delay distributions. 13
4 HIC MCV1 delay distributions. 14
5 Measles model schematic. 15
6 Comparison of transmissibility cutoff. 16
7 Measles vaccination implementation. 17
8 The mean age of measles infection for different R_0. 18
9 Additional optimization results. 19
10 Optimal age clusters. 20
11 Post-vaccination MAI and optimal MCV1 age. 21
12 Mantel tests between social contact matrices. 24
13 High burden age-group demographic type comparisons. 25
14 Times to converge to the optimal incidences. 26

1 Description of the model of measles transmission and vaccination

The measles model split the modeled population by disease state into susceptible (S), infected (I), recovered (R), and protected by maternal antibodies (M), along with a vaccinated-susceptible (VS) compartment to model people who received at least one dose of measles-containing vaccine (MCV) but failed to mount a protective immune response. The total population was defined as $N = S + I + R + M + VS$.

The model was also age-structured, splitting each disease state into age groups, resulting in the compartments M_i, S_i, I_i, R_i, and VS_i for each age group i. Hence, the total population in age group i was defined as $N_i = M_i + S_i + I_i + R_i + VS_i$. People aged out of each age group at a rate δ_i, determined by the width of the age group, and died with a mortality rate μ_i, determined by the demographic type. To facilitate the analysis, the population was split into monthly age groups between 0–59 months, then 5 yearly from 5 to 79 years, resulting in 75 age groups. As shown in Supplementary Figure 5, we modeled vaccination as occurring when populations transition between age groups.

1.1 Protected by maternal antibodies

Infants were born into M_1 at a rate pb, where b is the birth rate, and p is the proportion of infants born to protected mothers. Hence, p represents the prevalence of women of childbearing age who were recovered or successfully vaccinated against measles.

$$p = \frac{\sum_{i=63}^{i=71} R_i}{\sum_{i=63}^{i=71} N_i}. \tag{1}$$

Infants leave the compartment either when maternal antibody protection wanes at rate m, when they age out, or due to death. Hence, the equation describing the dynamics of the population protected by maternal antibodies compartment is

$$\frac{\mathrm{d}M_1}{\mathrm{d}t} = pb - mM_1 - \delta_1 M_1 - \mu_1 M_1, \tag{2}$$

for the first age group, and,

$$\frac{\mathrm{d}M_i}{\mathrm{d}t} = \delta_{i-1} M_{i-1} - mM_i - \delta_i M_i - \mu_i M_i, \tag{3}$$

for all older age groups.

1.2 Susceptible

Infants born to unprotected mothers entered the first susceptible compartment at a rate $(1 - p)b$. People also entered susceptible compartments by losing maternal antibodies and, for $i > 1$, by aging into the susceptible compartment from the age group before

without being vaccinated. People then left the susceptible compartment by aging out, dying, or being infected.

We modeled vaccination occurring at the transitions between age groups. Vaccination with MCV dose 1 (MCV1) occurred with probability $v_i^{(1)}$, and was successful with probability $VE_i^{(1)}$ (the age-dependent MCV1 vaccine effectiveness (VE)) and unsuccessful with probability $1 - VE_i^{(1)}$. The remaining infants aged into the next age group's susceptible compartment.

The probability of MCV1 vaccination was determined by the MCV1 delay distribution and overall MCV1 coverage and is equivalent to the probability of receiving MCV1 at age i given that MCV1 was not received at any prior age.

1.2.1 Incorporating vaccination

Let $V_i^{(k)}$ be the event that vaccination with MCV dose k occurs at age i, and $V^{(k)}$ the event that vaccination with MCV dose k occurs at all. Then, the delay distribution and the initial vaccination age ($i_0^{(k)}$) for MCV dose k gives the probability of vaccination with MCV dose k occurring at a given age i, given vaccination with MCV dose k occurs at all, $p_i^{(k)}$. Hence,

$$p_i^{(k)} = Pr(V_i^{(k)}|V^{(k)}). \tag{4}$$

We assume that vaccination with dose k occurs within 24 months of $i_0^{(k)}$. Hence, the probability that vaccination with MCV dose k occurs at age i becomes

$$\bar{p}_i^{(k)} = Pr(V_i^k) = \frac{\kappa_k p_i^{(k)}}{\sum_{i=i_0^{(k)}}^{i_0^{(k)}+24} p_i^{(k)}}, \tag{5}$$

where κ_k is the dose dependent MCV coverage, $Pr(V^{(k)})$.

For parameterization of our transmission model, we require $v_i^{(k)}$, the probability that vaccination with MCV dose k occurs at age i given that vaccination does not occur at ages $i_0^{(k)}$ to $i-1$. Let $!(V_{i_0:i-1}^{(k)})$ be the event that vaccination with dose k does not occur between ages $i_0^{(k)}$ and $i-1$. Hence, we require

$$v_i^k = Pr(V_i^k|!(V_{i_0:i-1}^{(k)})). \tag{6}$$

By Bayes' theorem, this becomes

$$v_i^{(k)} = \frac{Pr(!(V_{i_0:i-1}^{(k)})|V_i^k)Pr(V_i^k)}{Pr(!(V_{i_0:i-1}^{(k)}))}. \tag{7}$$

As $Pr(!(V^{(k)}_{i0:i-1}))$ is the probability of not being vaccinated by age $i-1$, this is equivalent to $1 - \sum_{i=i_0}^{i-1} \bar{p}_i^{(k)}$. Hence,

$$v_i^{(k)} = \frac{\bar{p}_i^{(k)}}{1 - \sum_{i=i_0}^{i-1} \bar{p}_i^{(k)}}. \tag{8}$$

1.2.2 Susceptible equations

Taken together, the equations for the susceptible compartments become

$$\frac{\mathrm{d}S_1}{\mathrm{d}t} = (1-p)b + mM_1 - \lambda_1 S_1 - \delta_1 S_1 - \mu_1 S_1, \tag{9}$$

for the first age group, and

$$\frac{\mathrm{d}S_i}{\mathrm{d}t} = mM_i + \delta_{i-1}(1 - v_{i-1}^{(1)})S_i - \lambda_i S_i - \delta_i S_i - \mu_i S_i, \tag{10}$$

for older age groups.

Here, the term $\lambda_i S_i$ models infection, where the age-dependent force of infection is

$$\lambda_i = \beta\big(q \sum_{j=1}^{60} \frac{c_{i,j} I_j}{N_j} + \sum_{j=61}^{75} \frac{c_{i,j} I_j}{N_j}\big). \tag{11}$$

β denotes the infection probability given contact, derived from the basic reproduction number R_0 using the next-generation matrix [1], q denotes the relative transmissibility of the < 5 years age group, and $c_{i,j}$ the per-capita frequency of contact between age groups i and j, determined by the social contact matrix (SCM).

1.3 Infected

People entered the infected compartment for a given age group either through infection from the susceptible compartment or the vaccinated-susceptible compartment in the same age group, or by aging into the compartment from the previous age group's infected compartment. People exited the compartment by recovering from measles at rate γ, dying, or aging out of the compartment. Hence, the equations describing the dynamics of the infected populations are

$$\frac{\mathrm{d}I_1}{\mathrm{d}t} = \lambda_1(S_1 + VS_1) - \gamma I_i - \delta_1 I_1 - \mu_1 I_1, \tag{12}$$

for the first age group, and

$$\frac{\mathrm{d}I_i}{\mathrm{d}t} = \lambda_i(S_i + VS_1) + \delta_{i-1} I_{i-1} - \gamma I_i - \delta_i I_i - \mu_i I_i, \tag{13}$$

for all other age groups.

1.4 Recovered

Individuals entered the recovered compartment or a given age group via four paths: by recovering from measles infection; by aging out of the previous age group's recovered compartment; by being successfully vaccinated with MCV1, occurring with rate $\delta_{i-1}v_{i-1}^{(1)}VE_{i-1}^{(1)}$; or, by being successfully vaccinated with MCV dose 2 (MCV2), following unsuccessful vaccination with MCV1, with rate $\delta_{i-1}v_{i-1}^{(2)}VE_{i-1}^{(2)}$. Recovered individuals then remained in the recovered compartment and aged into the next age group or died. The equations modeling the dynamics are, hence,

$$\frac{\mathrm{d}R_1}{\mathrm{d}t} = \gamma I_1 - \delta_1 R_1 - \mu_1 R_1, \tag{14}$$

for age group 1, and

$$\frac{\mathrm{d}R_i}{\mathrm{d}t} = \gamma I_i + \delta_{i-1}R_{i-1} + \delta_{i-1}v_{i-1}^{(1)}VE_{i-1}^{(1)}S_{i-1} + \delta_{i-1}v_{i-1}^{(2)}VE_{i-1}^{(2)}VS_{i-1} - \delta_i R_i - \mu_i R_i, \tag{15}$$

for all older age groups.

1.5 Vaccinated susceptible

Individuals entered an age group's vaccinated susceptible compartment either through unsuccessful vaccination with MCV1 with rate $\delta_{i-1}v_{i-1}^{(1)}(1-VE_{i-1}^{(1)})$, through unsuccessful vaccination with MCV2 with rate $\delta_{i-1}v_{i-1}^{(2)}(1-VE_{i-1}^{(2)})$, or by aging out of the prior vaccinated susceptible compartment without receiving MVC2, with rate $\delta_{i-1}(1-v_{i-1}^{(2)})$. People left the compartment either by being infected, by dying, by aging out without receiving MVC2, by receiving unsuccessful MCV2 vaccination, or by receiving successful MCV2 with rate $\delta_i v_i^{(2)} VE_i^{(2)}$. Hence, the equation for the first vaccinated susceptible age group is

$$\frac{\mathrm{d}VS_1}{\mathrm{d}t} = -\lambda_1 VS_1 - \delta_1 VS_1 - \mu_1 VS_1, \tag{16}$$

and,

$$\frac{\mathrm{d}VS_i}{\mathrm{d}t} = \delta_{i-1}v_{i-1}^{(1)}(1-VE_{i-1}^{(1)})S_{i-1} + \delta_{i-1}(1-v_{i-1}^{(2)}VE_{i-1}^{(2)})VS_{i-1} - \lambda_i VS_i - \delta_i VS_i - \mu_i VS_i, \tag{17}$$

for all older age groups.

2 Supplementary tables

Country	Population	Target	Quantile (%)	Delay	Years	Source
England	Black	365 days	25	26 days	2006-2014	[2]
England	Black	365 days	50	53 days	2006-2014	[2]
England	Black	365 days	75	115 days	2006-2014	[2]
England	White	365 days	25	23 days	2006-2014	[2]
England	White	365 days	50	44 days	2006-2014	[2]
England	White	365 days	75	79 days	2006-2014	[2]
Norway	All	489 days	70.7	31 days	2012	[3]
Norway	All	489 days	85.4	67 days	2012	[3]
Norway	All	489 days	92.7	205 days	2012	[3]
Switzerland	All	12 months	2.5	12.4 months	2005-2007	[4]
Switzerland	All	12 months	50	12.5 months	2005-2007	[4]
Switzerland	All	12 months	97.5	12.5 months	2005-2007	[4]
Switzerland	All	12 months	2.5	12.3 months	2008-2010	[4]
Switzerland	All	12 months	50	12.3 months	2008-2010	[4]
Switzerland	All	12 months	97.5	12.4 months	2008-2010	[4]
Switzerland	All	12 months	2.5	12.2 months	2011-2013	[4]
Switzerland	All	12 months	50	12.2 months	2011-2013	[4]
Switzerland	All	12 months	97.5	12.2 months	2011-2013	[4]
Switzerland	All	12 months	2.5	12.2 months	2014-2016	[4]
Switzerland	All	12 months	50	12.2 months	2014-2016	[4]
Switzerland	All	12 months	97.5	12.2 months	2014-2016	[4]
Switzerland	All	12 months	2.5	12.1 months	2017-2019	[4]
Switzerland	All	12 months	50	12.2 months	2017-2019	[4]
Switzerland	All	12 months	97.5	12.2 months	2017-2019	[4]

Table 1: Reported delays from high income countries, extracted from [2–4]. Note that different studies reported delays in different units, and for different quantiles.

Parameter	Interpretation	Value	Source
i	Age group	1–75	-
N	Total population size	10 million	-
δ_i	Age-specific aging rate	0.0005, 0.03 day^{-1}	-
μ_i	Age-specific mortality rate	Demographic type	-
p	Proportion of infants born to protected mothers	Equation 1	-
b	Birth rate	342.5 day^{-1}	-
$1/m$	Maternal antibody duration	2.9 months	[5]
k	MCV dose	1, 2	-
$v_i^{(k)}$	Age specific dose k conditional probability	Equation 8	-
$VE_i^{(k)}$	Age specific dose k VE	VE SCAM	[6]
$i_0^{(1)}$	Recommended MCV1 age	6–20 months	-
$i_0^{(2)}$	Recommended MCV2 age	$i_0^{(2)} = i_0^{(1)} + 6$	-
κ_1	MCV1 vaccine coverage	45%–85%	[7]
κ_2	MCV2 vaccine coverage	$\kappa_2 = \kappa_1 - 5\%$	[7]
λ_i	Age specific force of infection	Equation 11	-
$c_{i,j}$	Contact rate between age groups i and j	Values from SCMs	[8–10]
q	<5 years transmissibility	0.005–46.3	Fitted
R_0	Basic reproduction number	10–20	[11]
$1/\gamma$	Infectious period	13 days	[12]

Table 2: Model parameters used in the measles transmission and vaccination model.

3 Supplementary figures

Location	Age (years)	Time period	Transmission level
England and Wales	4.5 – 5.5	1944 – 1960	Low
Various localities in North America	4.0 – 6.0	1912 – 1918	Low
Zambia, Rhodesia, and South Africa	3.0 – 4.0	1960 – 1968	Medium
Nepal (Terai)	3.0 – 4.0	1977	Medium
Ghana	2.0 – 3.0	1960 – 1968	High
Eastern Nigeria	2.0 – 3.0	1960 – 1968	High
India (Pondicherry)	2.0 – 3.0	1978	High
Morocco	2.0 – 3.0	1960	High

Table 3: Historical pre-vaccine mean ages of measles infection (MAI) by location [13].

Fixed effects					
	Estimate	Standard Error	95% CI	t-value	F-value
Intercept	13.8	0.8	(12.2, 15.5)	17.3	
Low → medium	-1.7	0.02	(-1.6, -1.5)	-82.7	15537
Low → high	-3.7	0.02	(-3.7, -3.6)	-176.2	15537
**Random Effects**					
	Type		Variance	Standard deviation	
SCM	Intercept		2.9	1.7	
MCV1 coverage	Intercept		1.1	1.1	

Table 4: Coefficients of a linear mixed effects model of optimal age predicted by transmission level (15,398 observations). Model equation: Optimal age $\sim$ Transmission level $+ (1|\,\text{SCM}) + (1|\,\text{MCV1 coverage})$. Confidence intervals (CI) were calculated using likelihood ratio testing.

Fixed effects					
	Estimate	Standard Error	95% CI	t-value	F-value
Intercept	7.9	1.2	(5.1, 10.6)	6.352	
MCV1 coverage	0.06	0.0006	(0.06, 0.07)	103.2	10660
**Random Effects**					
	Type		Variance	Standard deviation	
SCM	Intercept		2.9	1.7	
Transmission level	Intercept		3.4	1.8	

Table 5: Coefficients of a linear mixed effects model of optimal age predicted by MCV1 coverage (15,398 observations). Model equation: Optimal age $\sim$ MCV1 coverage + (1| SCM) + (1| Transmission level).

Fixed effects					
	Estimate	Standard Error	95% CI	t-value	F-value
Intercept	6.6	0.05	(6.5, 6.7)	121.7	
MCV1 coverage	0.03	0.0008	(0.03, 0.03)	39.6	1566
**Random Effects**					
	Type		Variance	Standard deviation	
SCM	Intercept		0.001	0.03	

Table 6: Coefficients of a linear mixed effects model of optimal age predicted by MCV1 coverage, fitted to the results from the low- and lower-middle-income countries (LMIC) SCMs (6,000 observations). Model equation: Optimal age $\sim$ MCV1 coverage + (1| SCM).

Fixed effects					
	Estimate	Standard Error	95% CI	t-value	F-value
Intercept	8.6	0.2	(8.1, 9.1)	37.7	
Type 1 → Type 3	- 0.02	0.02	(-0.06, 0.03)	-0.7	0.5
**Random Effects**					
	Type		Variance	Standard deviation	
SCM	Intercept		0.001	0.03	
MCV1 coverage	Intercept		0.3	0.5	

Table 7: Coefficients of a linear mixed effects model of optimal age predicted by demography coverage, fitted to the results from the LMIC SCMs (6,000 observations). Model equation: Optimal age $\sim$ Demographic type + (1| SCM) + (1| MCV1 coverage).

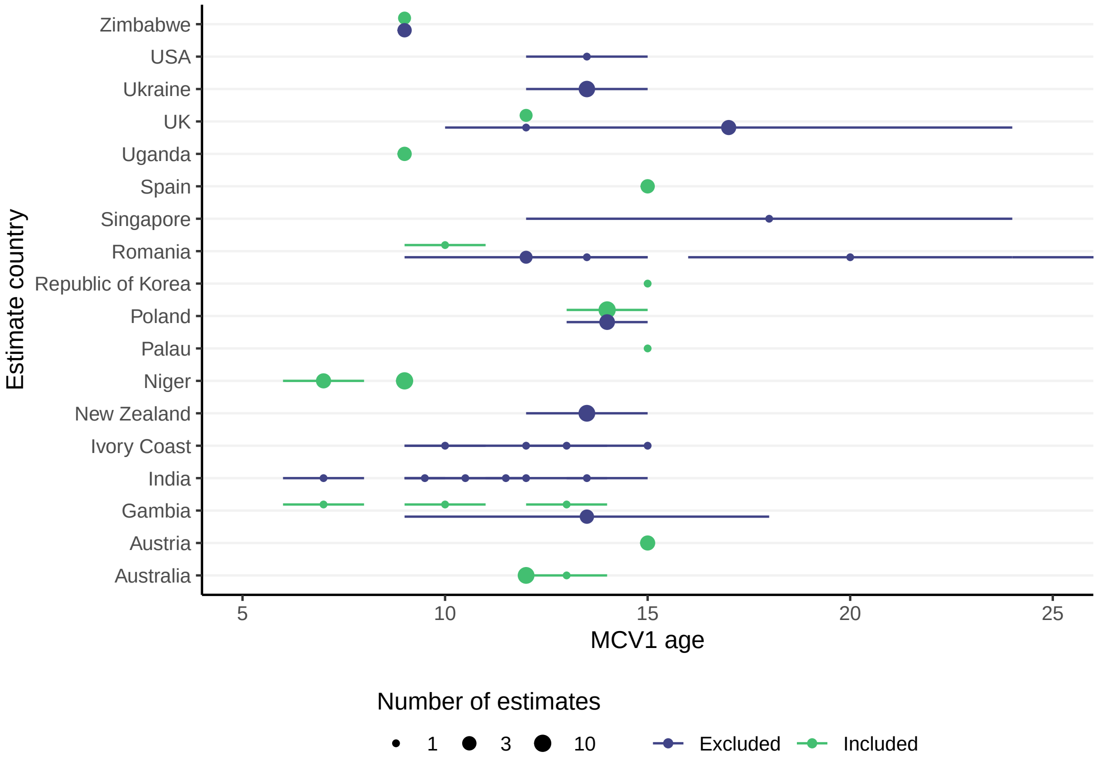

Figure 1: Vaccine effectiveness estimates (extracted from [6]) included and excluded from the analysis, according to estimate country. Not shown, 5 excluded estimates from Romania with MCV1 age between 24 and 132 months. Samples sizes for each estimate can be found in Supplementary data 2 of [6].

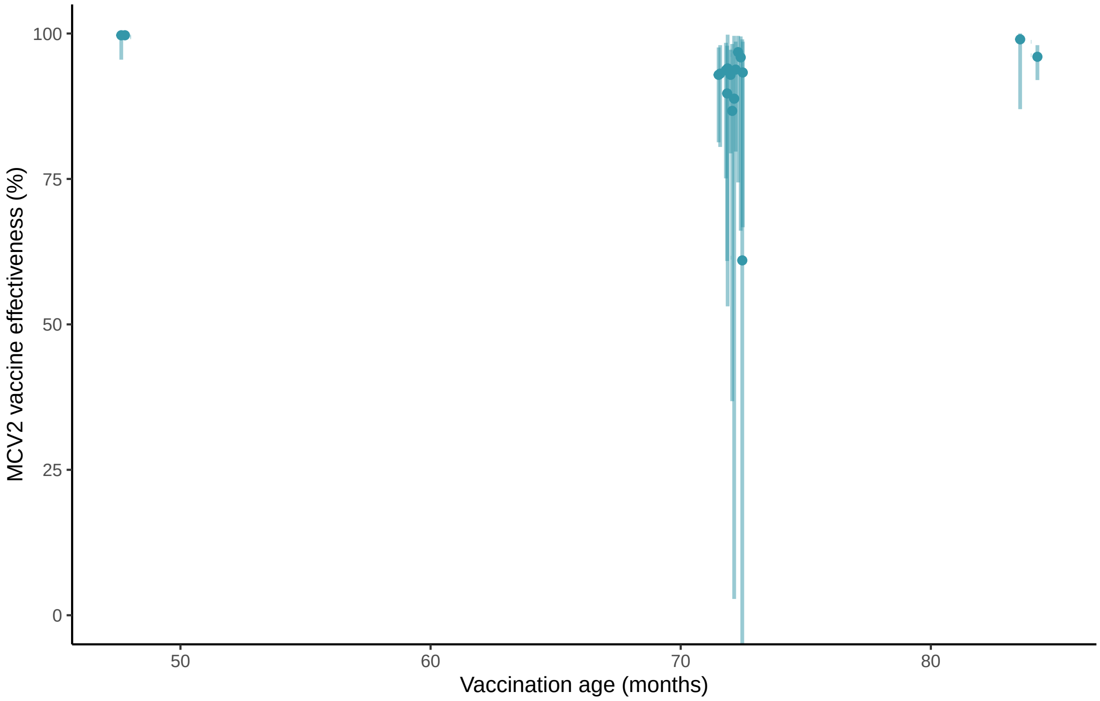

Figure 2: Estimates of vaccine effectiveness (VE) for MCV2, extracted from [6]. Points indicate the reported measure of VE, and horizontal lines give the 95% confidence interval. Estimates with MCV2 age range greater than 3 months are removed (6 estimates), leaving 17 VE estimates. Samples sizes for each estimate can be found in Supplementary data 2 of [6].

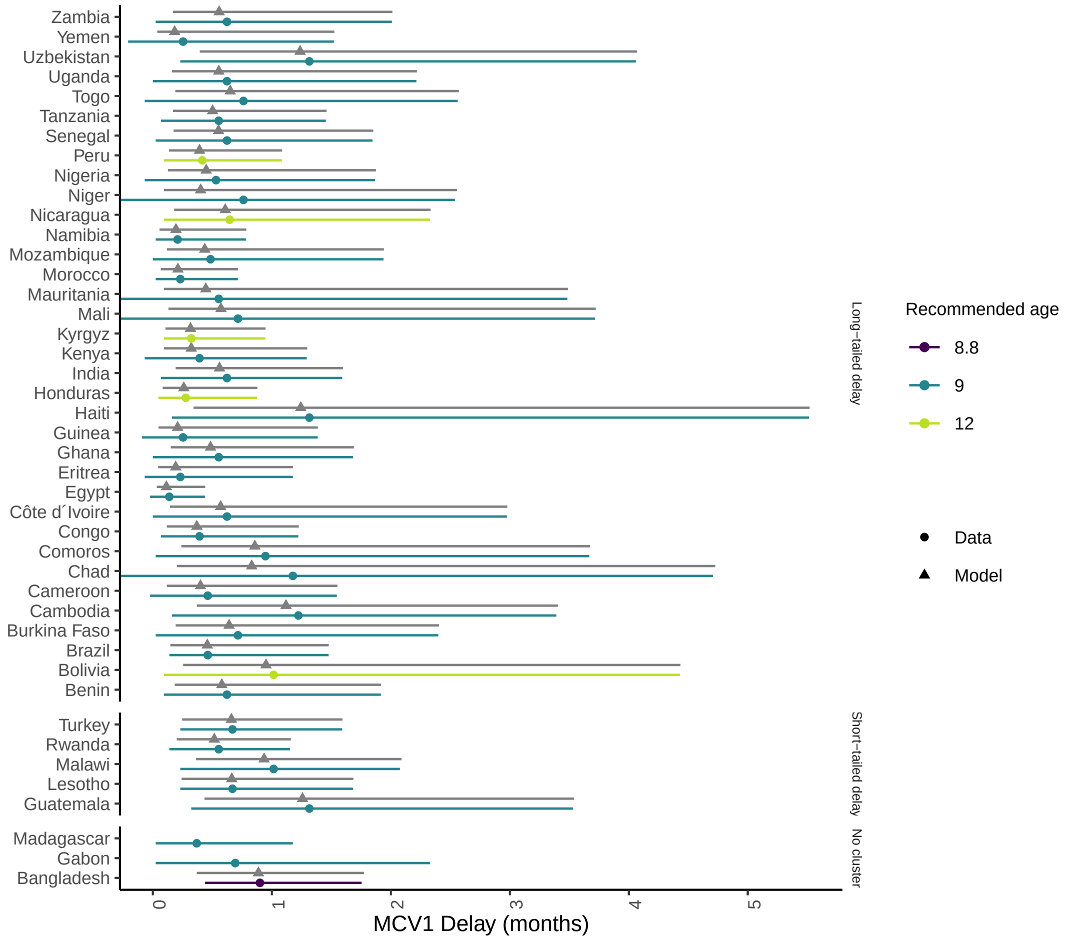

Figure 3: MCV1 delay data [14] and modeled Lomax distribution, grouped by cluster. Points indicate median delay and line segments indicate the 25% and 75% quantiles. Circles indicate data and triangles indicate modeled results.

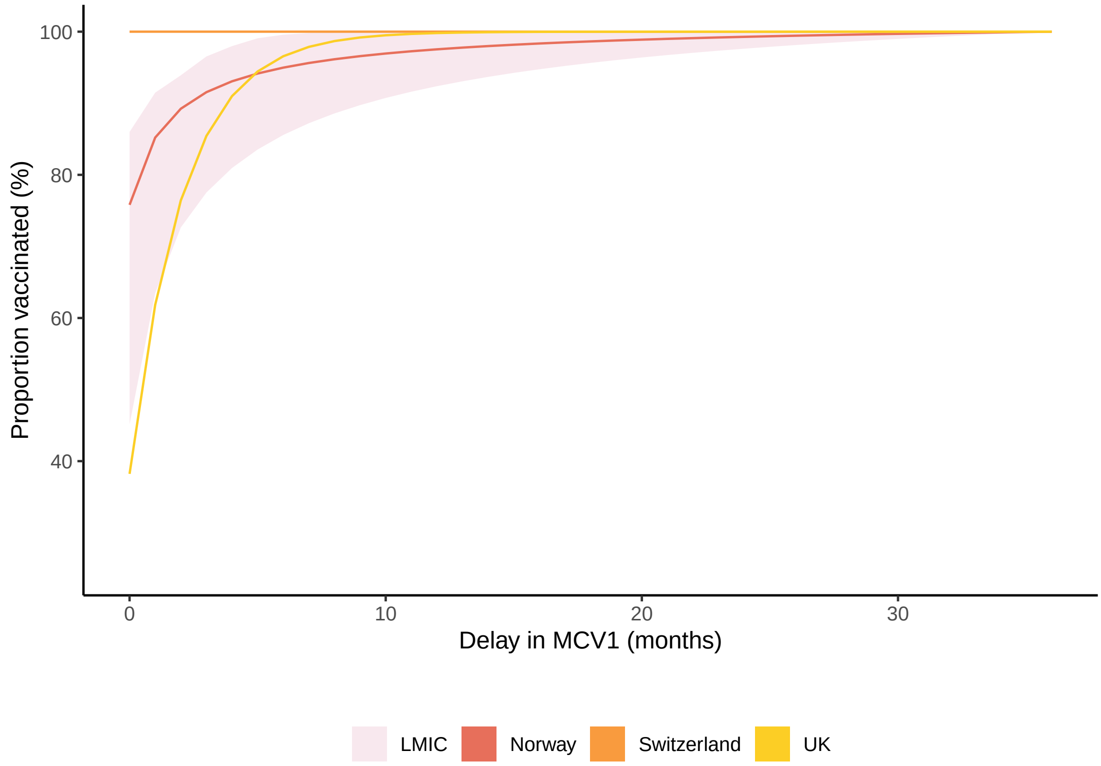

Figure 4: Modeled cumulative fraction vaccinated with MCV1 by month for 3 high-income countries (HIC) [2–4] compared to low- and middle-income countries [14]. The ribbon indicates the spread of fitted distributions from low- and middle-income countries. Curves indicate fitted Lomax distributions from high-income countries.

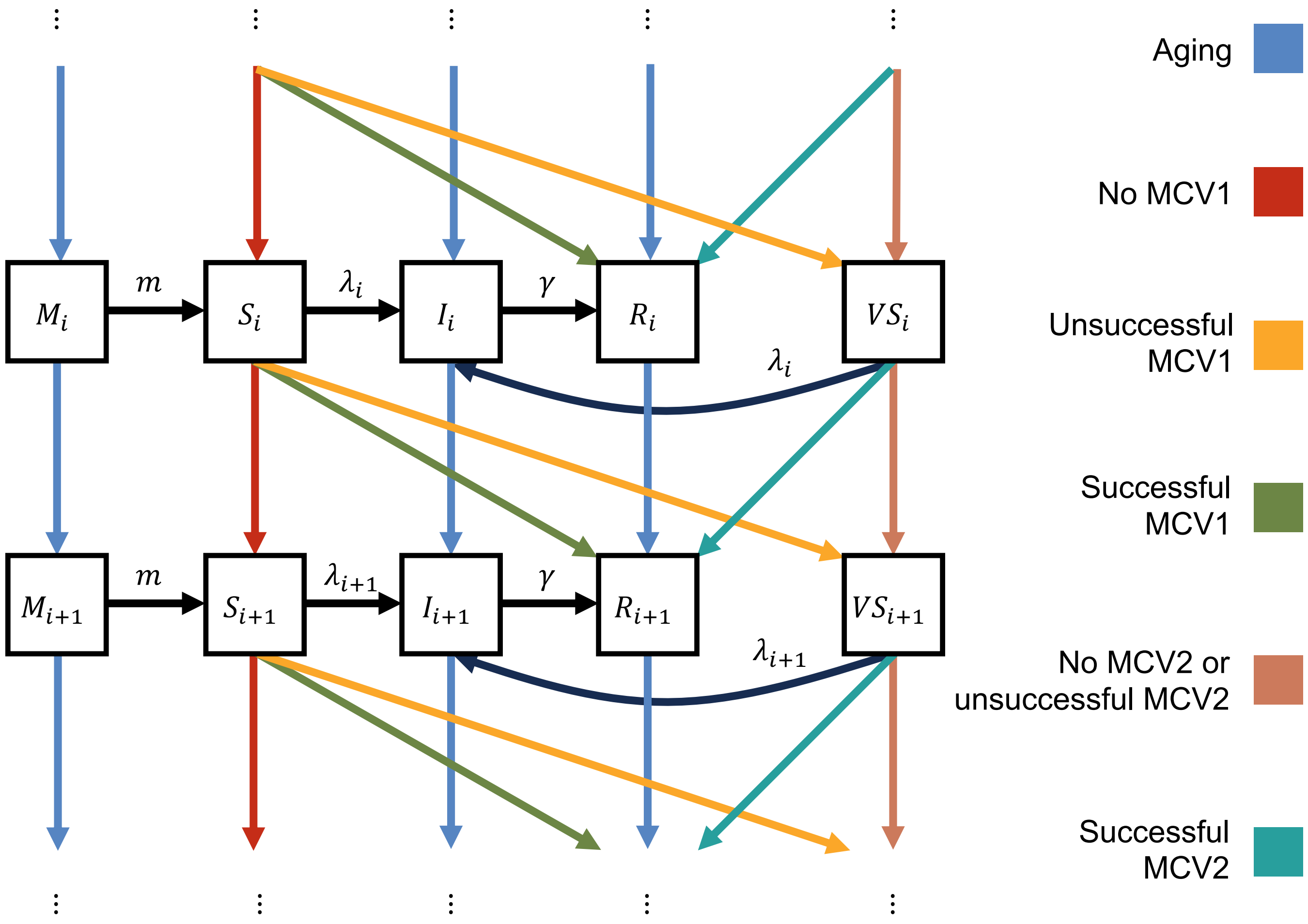

Figure 5: Model of measles transmission and vaccination schematic. Colored arrows indicate vaccination-related transitions occurring between age groups. Black arrows indicate transmission-related transitions occurring within age groups. For clarity, mortality is not shown.

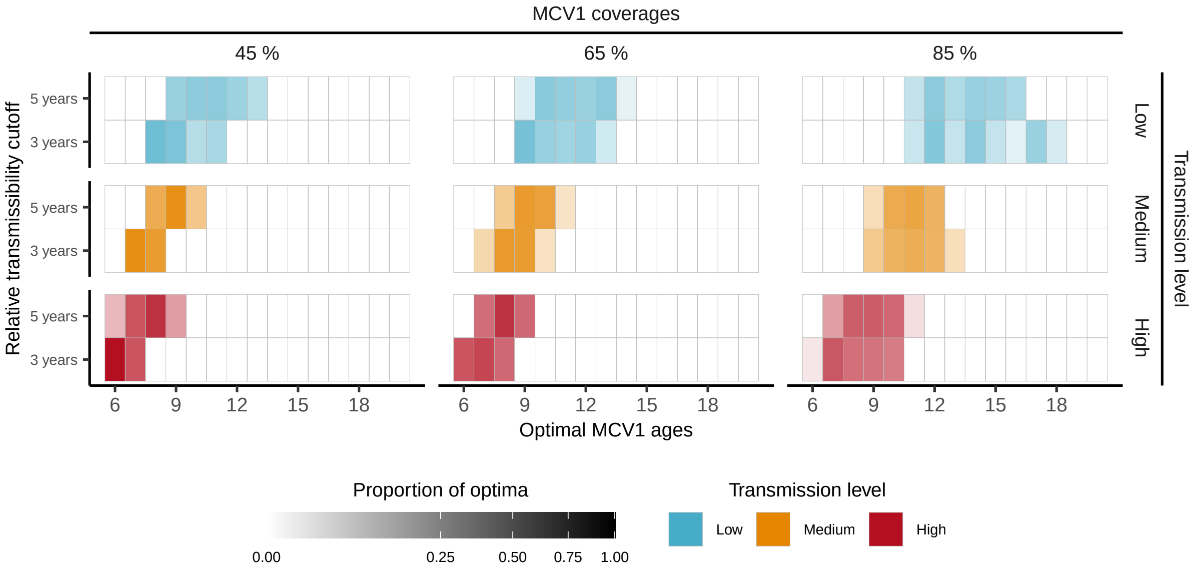

Figure 6: Heatmap comparing the optimal ages for the South Africa SCM when the relative transmissibility parameter is defined as < 3 years versus < 5 years. Opacity indicates the proportion of parameter sets with an optimum at a given MCV1 age. For clarity, the results for 55% and 75% vaccination coverage are not displayed.

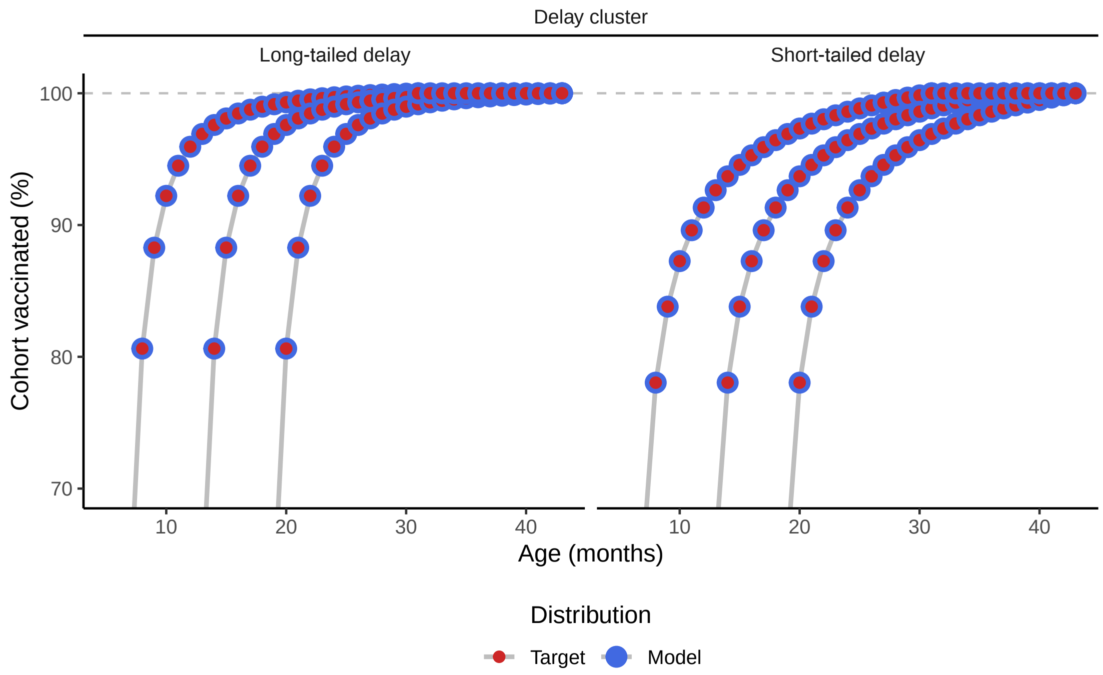

Figure 7: Comparison of the percentage of the population vaccinated with MCV1 by a given age according to the target distribution, derived from the delay distribution, and according to the measles transmission and vaccination model (detailed in 1.2.1).

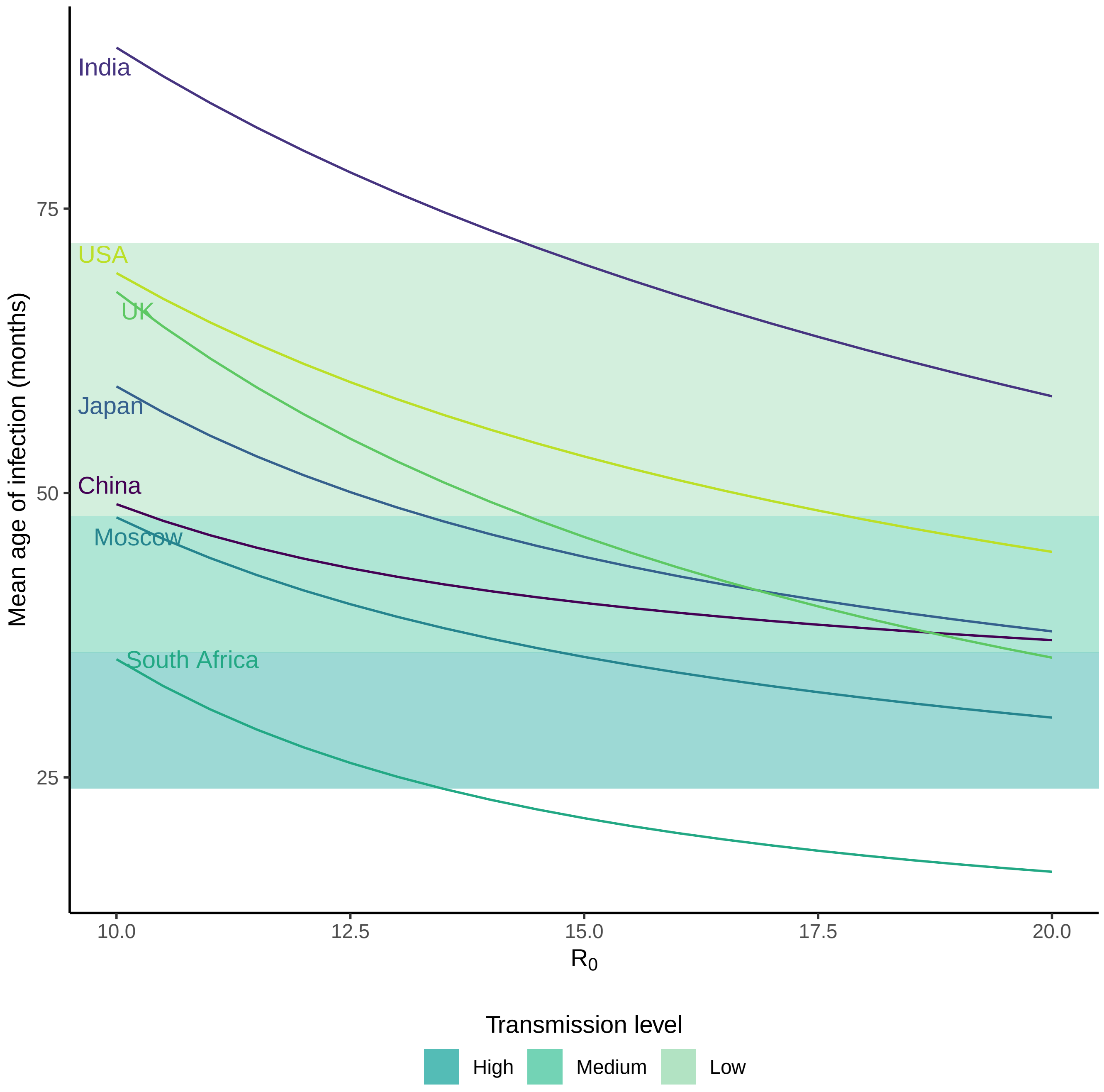

Figure 8: Modeled MAI for different SCMs, at typical reported values of R_0 [11]. The MAI values were calculated using the model with relative <5 years transmissibility, $q = 1$. Transmission levels were defined using historical reports of the MAI[13].

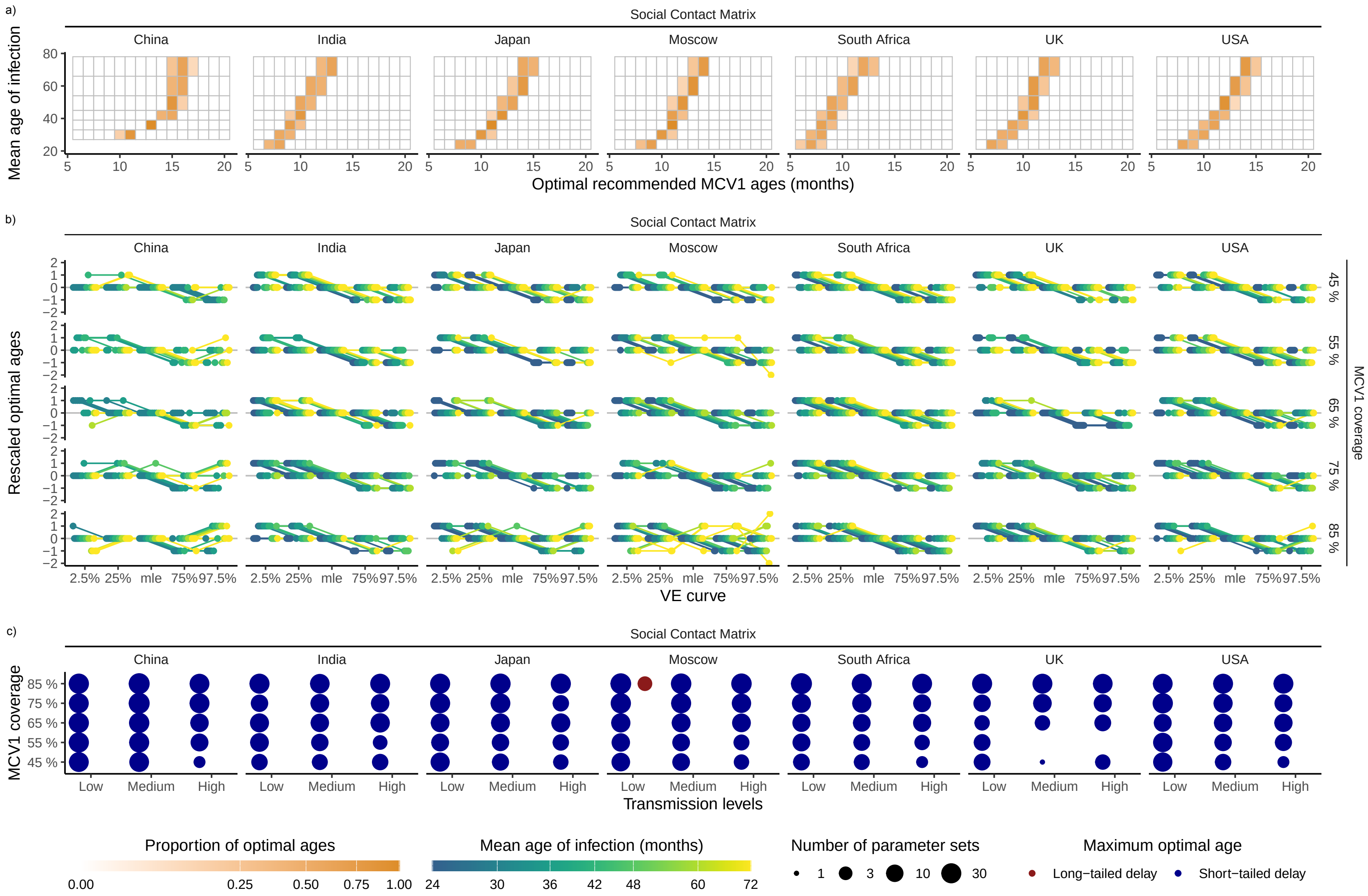

Figure 9: a) Impact of the mean age of infection on the optimal MCV1 age for each SCM. b) Impact of MCV1 VE curve on the optimal MCV1 age. Only parameter sets where changing the VE curve resulted in changes in optimal age are plotted. c) Impact of vaccination delay distribution on the optimal age. Only parameter sets with changes in optimal age are plotted.

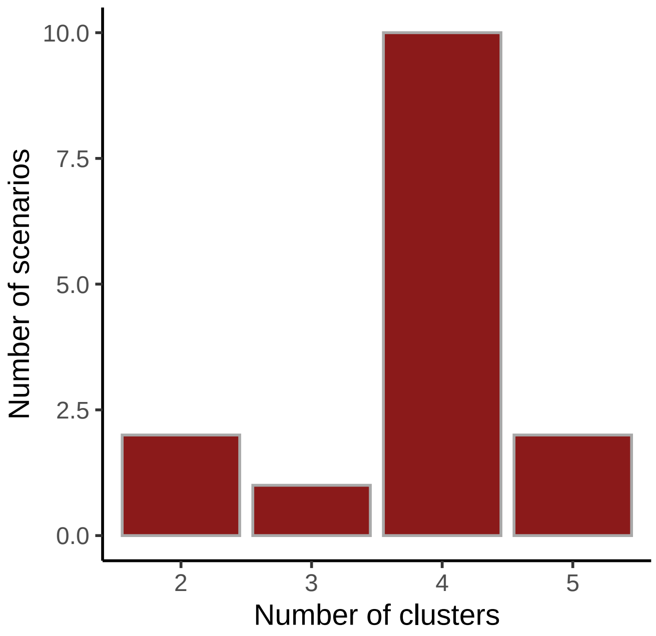

Figure 10: Histogram of the distribution of the number of optimal age clusters. For each MCV coverage-transmission level pair (scenario), the optimal MCV1 ages were clustered according to SCM, using Partitioning Around Medoids clustering with the number of clusters determined using the silhouette method [15].

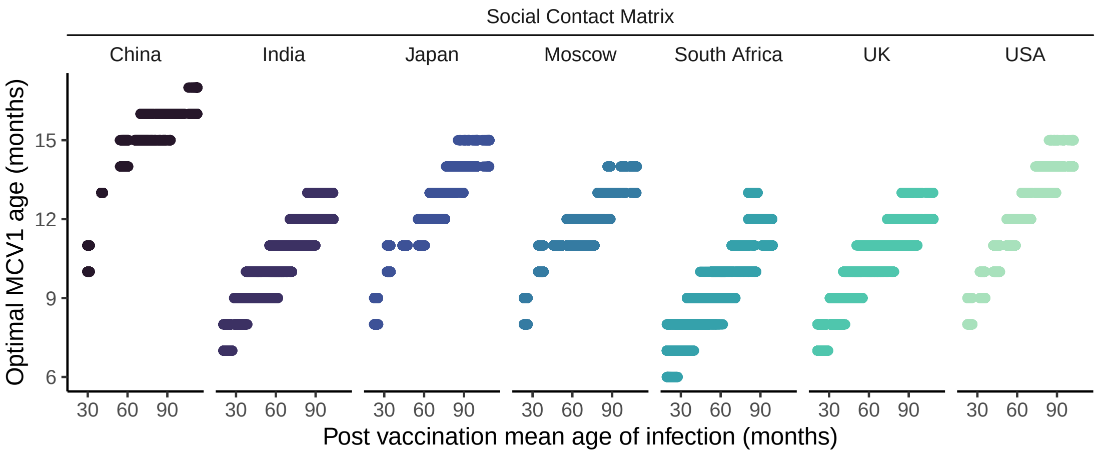

Figure 11: Optimal MCV1 age by post-vaccination MAI at 45% MCV1 coverage. The optimal age of a simulated parameter set increases with post-vaccination MAI across SCM. Data are from simulations at non-optimal MCV1 ages, compared against the corresponding optimal age.

References

1. Diekmann, O., Heesterbeek, J. A. P. & Metz, J. A. J. On the definition and the computation of the basic reproduction ratio R0 in models for infectious diseases in heterogeneous populations. *Journal of Mathematical Biology* **28,** 365–382. ISSN: 1432-1416. https://doi.org/10.1007/BF00178324 (June 1990).
2. Suffel, A. M., Walker, J. L., Williamson, E., McDonald, H. I. & Warren-Gash, C. Timeliness of childhood vaccination in England: A population-based cohort study. *Vaccine* **41,** 5775–5781. https://doi.org/10.1016/j.vaccine.2023.08.002 (Sept. 2023).
3. Riise, Ø. R. *et al.* Monitoring of timely and delayed vaccinations: a nation-wide registry-based study of Norwegian children aged $<$ 2 years. *BMC Pediatrics* **15,** 180. https://doi.org/10.1186/s12887-015-0487-4 (Nov. 2015).
4. Baroutsou, V. *et al.* National and regional variations in timely adherence to recommended measles vaccination scheme in 2-years old in Switzerland, 2005–2019. *Vaccine* **40,** 3055–3063. https://doi.org/10.1016/j.vaccine.2022.04.008 (May 2022).
5. Goeyvaerts, N., Leuridan, E., Faes, C., Van Damme, P. & Hens, N. Multi-disease analysis of maternal antibody decay using non-linear mixed models accounting for censoring. *Statistics in Medicine* **34,** 2858–2871. https://doi.org/10.1002/sim.6518 (Apr. 2015).
6. Hughes, S. L. *et al.* The effect of time since measles vaccination and age at first dose on measles vaccine effectiveness – A systematic review. *Vaccine* **38,** 460–469. https://doi.org/10.1016/j.vaccine.2019.10.090 (Jan. 2020).
7. World Health Organisation. *Comparison of Immunization coverage for Measles vaccination coverage and Incidence for Measles* 2022. %7Bhttps://immunizationdata.who.int/compare?GROUP=Countries&COMPARISON=type1__WIISE/MT_AD_COV_LONG+type2__WIISE/MT_AD_INC_RATE_LONG+option1__MCV_coverage+option2__MEASLES_incidence&YEAR=%7D.
8. Mistry, D. *et al.* Inferring high-resolution human mixing patterns for disease modeling. *Nature Communications* **12,** 323. https://doi.org/10.1038/s41467-020-20544-y (Jan. 2021).
9. Mossong, J. *et al.* Social Contacts and Mixing Patterns Relevant to the Spread of Infectious Diseases. *PLOS Medicine* **5,** e74. https://doi.org/10.1371/journal.pmed.0050074 (Mar. 2008).
10. Prem, K. *et al.* Projecting contact matrices in 177 geographical regions: An update and comparison with empirical data for the COVID-19 era. *PLOS Computational Biology* **17,** e1009098. https://doi.org/10.1371/journal.pcbi.1009098 (July 2021).

11. Guerra, F. M. *et al.* The basic reproduction number (R0) of measles: a systematic review. *The Lancet Infectious Diseases* **17,** e420–e428. https://doi.org/10.1016/S1473-3099(17)30307-9 (Dec. 2017).

12. Verguet, S. *et al.* Controlling measles using supplemental immunization activities: A mathematical model to inform optimal policy. *Vaccine* **33,** 1291–1296. http://dx.doi.org/10.1016/j.vaccine.2014.11.050 (Mar. 2015).

13. Anderson, R. & May, R. Vaccination against rubella and measles: Quantitative investigations of different policies. *The Journal of Hygiene* **90,** 259–325. https://doi.org/10.1017/S002217240002893X (May 1983).

14. Clark, A. & Sanderson, C. Timing of children's vaccinations in 45 low-income and middle-income countries: an analysis of survey data. *The Lancet* **373,** 1543–1549. https://doi.org/10.1016/S0140-6736(09)60317-2 (May 2009).

15. Kaufman, L. & Rousseeuw, P. J. *Finding Groups in Data* ISBN: 9780470316801. https://doi.org/10.1002/9780470316801 (John Wiley & Sons, Inc., New Jersey, 2005).

16. Legendre, P. & Legendre, L. *Numerical Ecology* 3rd ed. ISBN: 978-0-444-53869-7 (Elsevier, July 2012).

17. Demetrius, L. Adaptive value, entropy and survivorship curves. *Nature* **275,** 213–214. https://doi.org/10.1038/275213a0 (Sept. 1978).

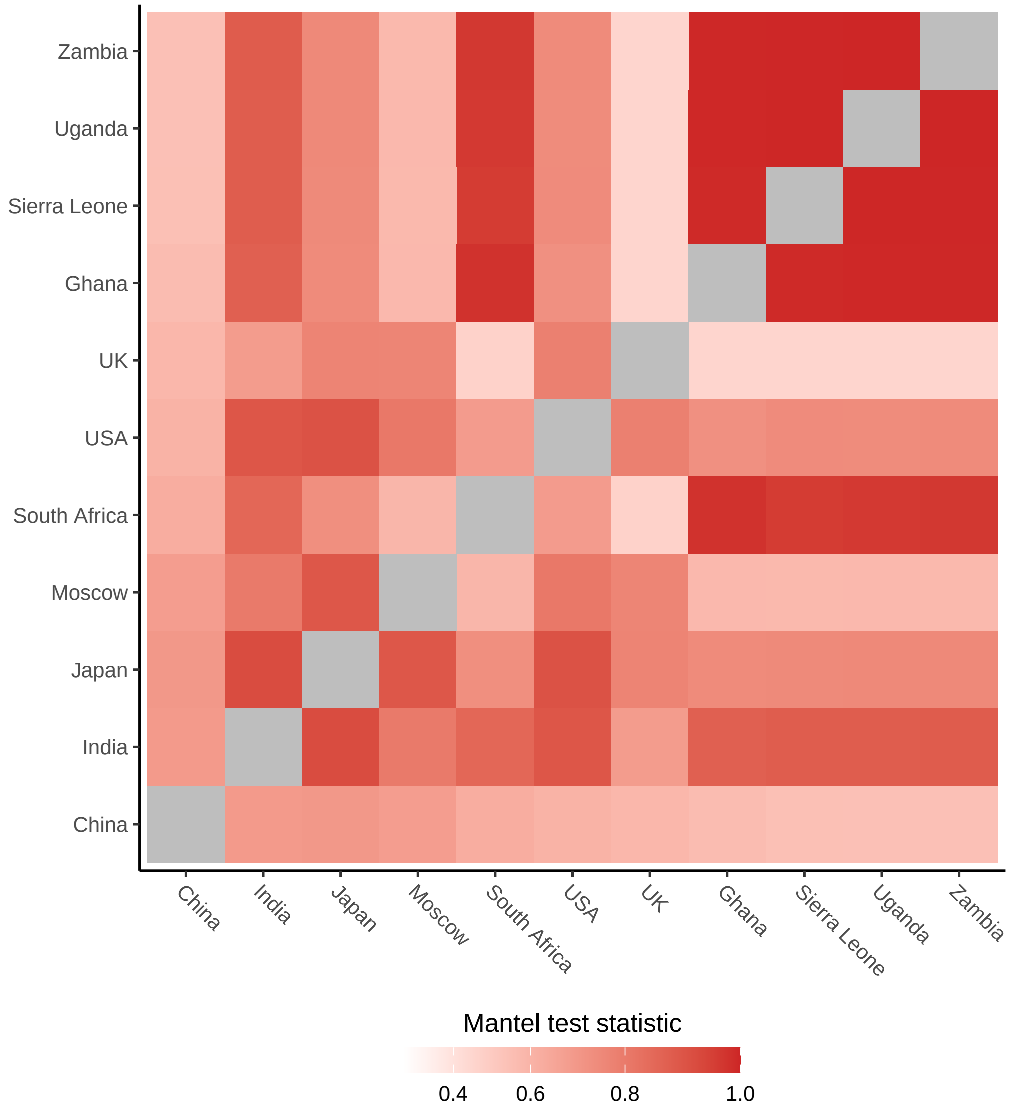

Figure 12: Heatmap of Pearson Mantel test statistics [16] between pairs of SCMs. Mantel tests were performed on SCMs corrected for type 1 age-demography [17], projected to the model age groups. For all SCM pairs, the asymptotic significance level was 0.001. African SCMs (South Africa, Ghana, Sierra Leone, Uganda, Zambia) are shown to have higher mantel statistics with one another than with other SCMs.

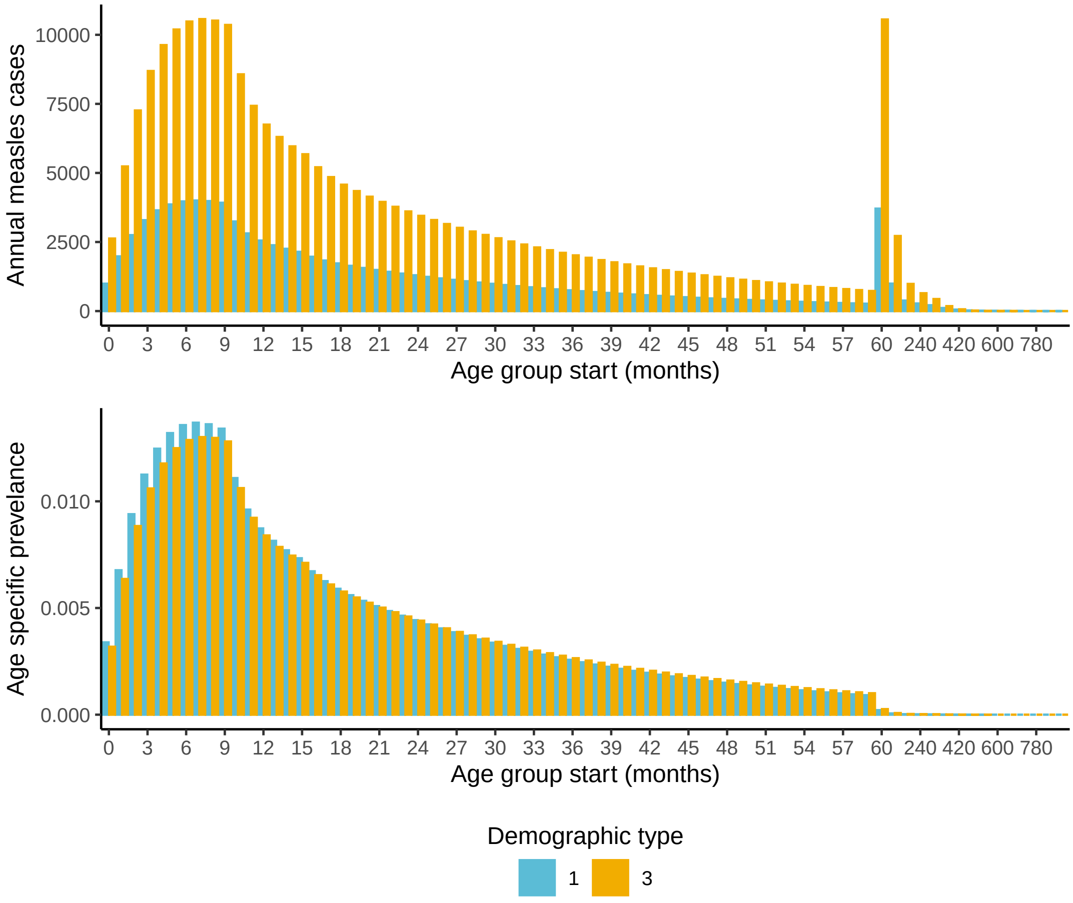

Figure 13: Simulated measles cases and prevalence by age group, in an example scenario, with the Ghanaian SCM, 45% MCV1 coverage, and MCV1 scheduled at 9 months, for each demographic type. To keep the pre-vaccine MAI equal (24 months), the R_0 and q parameters vary slightly between demographic types. Note that the axes' scales change after 60 months.

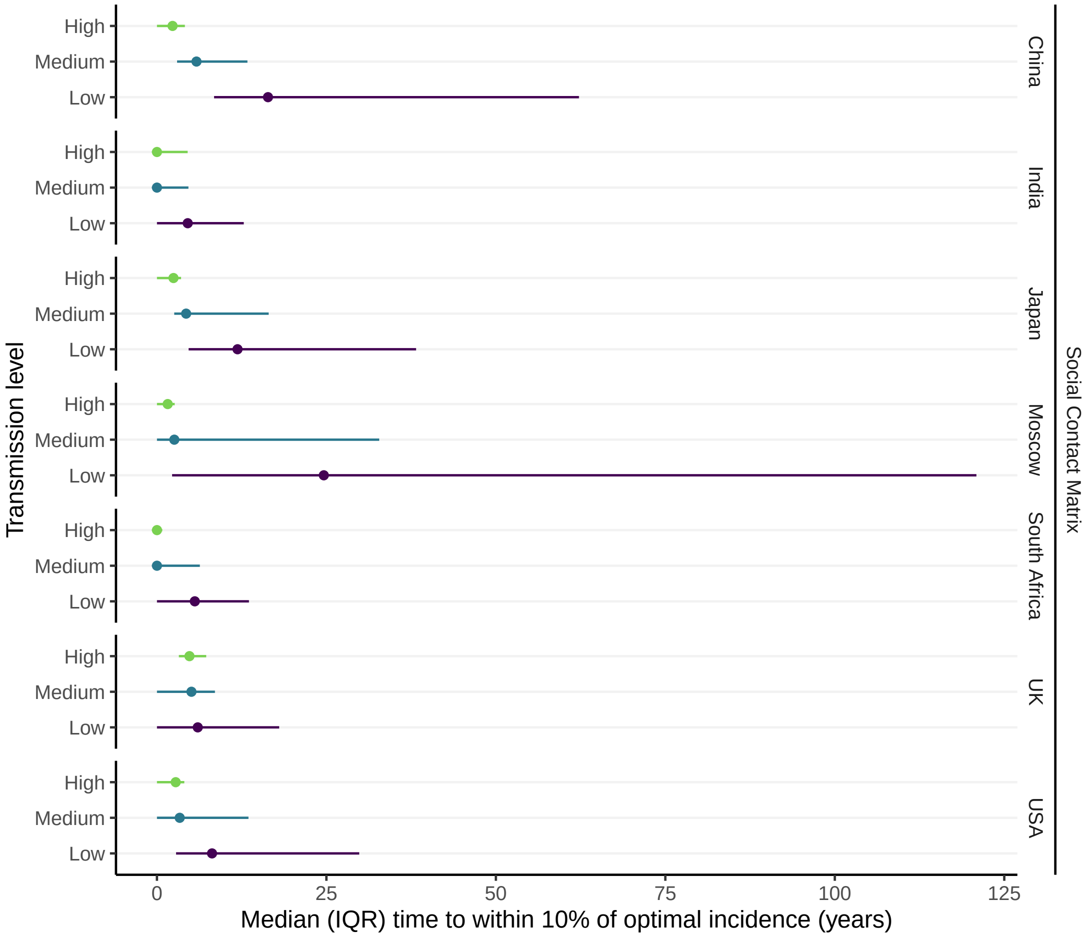

Figure 14: The times to converge to within 10% of the optimal incidence from 9 or 12 months by SCM and transmission level. Dots indicate the median time to converge, and lines indicate the interquartile range (IQR).
