# Supplementary material for: Estimating the optimal age for infant measles vaccination
Source: Nat Commun. 2024 Nov 15;15:9919. doi: 10.1038/s41467-024-53415-x (PMC11568136; doi:10.1038/s41467-024-53415-x)
Supplement: Supplementary file 2 — Reporting Summary [file 41467_2024_53415_MOESM2_ESM.pdf]

nature portfolio

Corresponding author(s): Elizabeth Goult

Last updated by author(s): Oct 9, 2024

Reporting Summary

Nature Portfolio wishes to improve the reproducibility of the work that we publish. This form provides structure for consistency and transparency in reporting. For further information on Nature Portfolio policies, see our Editorial Policies and the Editorial Policy Checklist.

Statistics

For all statistical analyses, confirm that the following items are present in the figure legend, table legend, main text, or Methods section.

n/a	Confirmed	
☐	☒	The exact sample size (*n*) for each experimental group/condition, given as a discrete number and unit of measurement
☒	☐	A statement on whether measurements were taken from distinct samples or whether the same sample was measured repeatedly
☐	☒	The statistical test(s) used AND whether they are one- or two-sided<br>*Only common tests should be described solely by name; describe more complex techniques in the Methods section.*
☐	☒	A description of all covariates tested
☐	☒	A description of any assumptions or corrections, such as tests of normality and adjustment for multiple comparisons
☐	☒	A full description of the statistical parameters including central tendency (e.g. means) or other basic estimates (e.g. regression coefficient) AND variation (e.g. standard deviation) or associated estimates of uncertainty (e.g. confidence intervals)
☐	☒	For null hypothesis testing, the test statistic (e.g. *F*, *t*, *r*) with confidence intervals, effect sizes, degrees of freedom and *P* value noted<br>*Give P values as exact values whenever suitable.*
☒	☐	For Bayesian analysis, information on the choice of priors and Markov chain Monte Carlo settings
☒	☐	For hierarchical and complex designs, identification of the appropriate level for tests and full reporting of outcomes
☒	☐	Estimates of effect sizes (e.g. Cohen's *d*, Pearson's *r*), indicating how they were calculated

Our web collection on statistics for biologists contains articles on many of the points above.

Software and code

Policy information about availability of computer code

Data collection	No unpublished data was collected for this study.
Data analysis	Analysis was carried out using R version 4.1.1, using the R package tidyverse version 1.3.1. Partial correlations were calculated using the package ppcor version 1.1. SCAMs were fitted using the package scam version 1.2.12. Lomax distributions were fitted using the algorithm L-BFGS-B. PAM clustering was carried out using the R packages cluster version 2.1.2 and factoextra version 1.0.7. Mantel tests were calculated using the R package vegan version 2.6.2, using Pearson correlation. The measles model was implemented in C and R, using the R package pomp version 3.6. Parameter fitting was carried out using the subplex algorithm in the R package nloptr version 2.0.0. Mixed-effect models were fitted using the package lme4 version 1.1.27.1. Figures were created using the R package ggplot2 version 3.5.0, with country boundaries in Figure 2 taken from the Natural Earth 1:50m world map, version 2.0.<br><br>Processed data and code are deposited in the Open Research Data Repository of the Max Planck Society, Edmond (https://doi.org/10.17617/3.50X626) with no end date.

For manuscripts utilizing custom algorithms or software that are central to the research but not yet described in published literature, software must be made available to editors and reviewers. We strongly encourage code deposition in a community repository (e.g. GitHub). See the Nature Portfolio guidelines for submitting code & software for further information.

Data

Policy information about availability of data

All manuscripts must include a data availability statement. This statement should provide the following information, where applicable:

- Accession codes, unique identifiers, or web links for publicly available datasets
- A description of any restrictions on data availability
- For clinical datasets or third party data, please ensure that the statement adheres to our policy

All data used are from published articles and published reports. Sources for measles incidence, recommended MCV1 age, and income group classification are included in Methods: Data on measles incidence, recommended MCV1 age, and income group classification. Details of the MCV vaccine effectiveness data are given in Methods: The relationship between MCV age and vaccine effectiveness. Details of MCV1 delay distribution data are included in Methods: The distribution of MCV1 delay. Sources for the social contact matrices are included in Methods: Recapitulating reported pre-vaccine mean ages of measles infection and Methods: Optimizing the MCV1 ages for social contact matrices from low- and lower-middle-income countries, which also details the sources for the age-group population sizes. Finally, details of MCV coverage sources are included in Methods: The optimal age to recommend MCV1.

Research involving human participants, their data, or biological material

Policy information about studies with human participants or human data. See also policy information about sex, gender (identity/presentation), and sexual orientation and race, ethnicity and racism.

Reporting on sex and gender	This is a simulation study with no human participants.
Reporting on race, ethnicity, or other socially relevant groupings	NA
Population characteristics	NA
Recruitment	NA
Ethics oversight	NA

Note that full information on the approval of the study protocol must also be provided in the manuscript.

Field-specific reporting

Please select the one below that is the best fit for your research. If you are not sure, read the appropriate sections before making your selection.

☒ Life sciences ☐ Behavioural & social sciences ☐ Ecological, evolutionary & environmental sciences

For a reference copy of the document with all sections, see nature.com/documents/nr-reporting-summary-flat.pdf

Life sciences study design

All studies must disclose on these points even when the disclosure is negative.

Sample size	This is a simulation study. Hence, the full simulated population (10 million) was sampled.
Data exclusions	Data on vaccine effectiveness were excluded if the vaccination age interval was >3 months. Data on vaccination delays from two countries were excluded as the delays were biased by measures to tackle ongoing measles outbreaks during the sampling period. Simulation results were excluded if the simulation failed to fulfill our convergence criteria. Full details on exclusions are included in our methods.
Replication	This is a simulation study. The model used is fully deterministic, so no replication was required. Results can be replicated by rerunning the code deposited at https://doi.org/10.17617/3.50X626.
Randomization	This is a simulation study, hence randomization is not relevant.
Blinding	This is a simulation study, so no blinding was required.

Reporting for specific materials, systems and methods

We require information from authors about some types of materials, experimental systems and methods used in many studies. Here, indicate whether each material, system or method listed is relevant to your study. If you are not sure if a list item applies to your research, read the appropriate section before selecting a response.

Materials & experimental systems

n/a	Involved in the study
☒	☐ Antibodies
☒	☐ Eukaryotic cell lines
☒	☐ Palaeontology and archaeology
☒	☐ Animals and other organisms
☒	☐ Clinical data
☒	☐ Dual use research of concern
☒	☐ Plants

Methods

n/a	Involved in the study
☒	☐ ChIP-seq
☒	☐ Flow cytometry
☒	☐ MRI-based neuroimaging

Plants

Seed stocks	*Report on the source of all seed stocks or other plant material used. If applicable, state the seed stock centre and catalogue number. If plant specimens were collected from the field, describe the collection location, date and sampling procedures.*
Novel plant genotypes	*Describe the methods by which all novel plant genotypes were produced. This includes those generated by transgenic approaches, gene editing, chemical/radiation-based mutagenesis and hybridization. For transgenic lines, describe the transformation method, the number of independent lines analyzed and the generation upon which experiments were performed. For gene-edited lines, describe the editor used, the endogenous sequence targeted for editing, the targeting guide RNA sequence (if applicable) and how the editor was applied.*
Authentication	*Describe any authentication procedures for each seed stock used or novel genotype generated. Describe any experiments used to assess the effect of a mutation and, where applicable, how potential secondary effects (e.g. second site T-DNA insertions, mosiacism, off-target gene editing) were examined.*
